# Characterization of blood donors and non-blood donors in Germany using an online survey

**DOI:** 10.1007/s12553-021-00532-y

**Published:** 2021-03-02

**Authors:** Benita Stock, Luis Möckel

**Affiliations:** 1 HSD Hochschule Döpfer GmbH, University of Applied Sciences Regensburg, Prüfeninger Straße 20, 93049 Regensburg, Germany; 2 HSD Hochschule Döpfer GmbH, University of Applied Sciences Cologne, Waidmarkt 3 & 9, 50676 Cologne, Germany

**Keywords:** Blood donation, Blood demand, Transfusion, Health service, Medical aid

## Abstract

Objectives of this study were to analyze characteristics influencing blood donation status, to identify anxieties and reasons for (non-)blood donation, and potential channels for future blood donation campaigns. A random population from Germany was interviewed using the online survey tool SoSci Survey. The access link to the questionnaire was distributed via snowball system and the Bavarian Red Cross. Statistical analysis was performed to identify factors influencing blood donation status. A total of 682 participants (27.3% blood donors) with a mean age of 33.4 and a standard deviation (SD) of 12.0 years were included into the analysis. Strongest factor associated with being blood donor was having a blood donor within family and friends (Odds ratio [OR]: 5.05 [95% confidence interval [95% CI]: 2.63; 9.70]; *p*≤0.001), whereas having anxiety related to blood donation was the strongest factor for being non-blood donor (OR: 0.11 [95% CI: 0.05; 0.21] *p*≤0.001). Other factors significantly influencing blood donor status were age, health-related quality of life, knowledge on blood donation, being an organ donor and having pre-conditions. Main anxieties avoiding blood donation were fear of physical consequences, and fear of the injection needle. Most frequently mentioned channels which should be used for blood donation campaigns were Instagram and free TV. Involving blood donors into campaigns to recruit new blood donors from their personal environment and to focus campaign content on physical benefits of blood donations might help to recruit new blood donors. In addition, running campaigns stronger on channels such as Instagram might increase their scope.

## Introduction

Blood donations are essential for different health care services such as the treatment of emergency patients and several kinds of surgeries. Between 2010 and 2019 the number of blood donations decreased by 12 % from 7.5 Mio. to 6.6 Mio. in Germany [1]. Previous publications have reported concerns on the maintenance of blood supply and potential blood shortages [2, 3]. A recent study by Greinacher et al. (2017) conducted in a north-eastern region of Germany showed a decline in blood donations in younger age groups between 2005 and 2015. Donation rates per 1,000 inhabitants decreased by 10.6% (140.5 to 125.6) and 28.1% (103.0 to 74.1) in the populations aged 18-30 and 31-40 years, respectively. In contrast, rates increased in the populations aged 51-60 years (75.5 to 88.4) by 17.1% and 61-69 years (22.5 to 51.2) by 127.5% [4]. Nevertheless, the authors discussed a further reduction in blood donations when the population aged 51-60 years is not able to donate blood anymore [4]. In addition, a prediction for the southern German region Baden-Wuerttemberg indicates a decrease in blood donation of 11% by 2030 compared to 2007, and a concurrent increase in blood product consumption of 10% in tertiary care centers and up to three times in primary care centers [5].

For the planning of blood donation campaigns and to stop the reduction in blood donations it is key to understand factors influencing blood donation status. A study with 542 undergraduate students in Hong Kong indicated that among other factors, gender, age, being an organ donor, knowledge on blood donation, and the self-reported health status were significantly associated with blood donation status [6]. A second study performed with 5640 (non-)blood donors in Germany showed that being blood donor is related to characteristics such as being female, of younger age, and more satisfied with the own health as well as being helpful [7].

But to the best of our knowledge currently missing for Germany are information on how exercise behavior, anxieties, education on blood donation, and especially how family and friends are influencing the blood donation status. Furthermore, it is key to understand which are preferred channels of (non-)blood donors in terms of promotion of blood donation campaigns. Therefore, the aim of this study was to characterize (non-)blood donors in Germany to gather further information on factors affecting blood donation status. Additionally, to understand reasons and anxieties influencing blood donation status and channels which could be used to promote future blood donation campaigns.

## Methods

### Study design

This study was a survey study on blood donation of a random population in Germany conducted between June 19th to August 28th, 2020. The survey was performed using the SoSci Survey online tool [8] and the link to access the survey was distributed via snowball sampling. Therefore, participants were instructed to distribute the link to the survey within their social environment. In addition, the access link was provided to the Bavarian Red Cross (BRK), a blood donation service, to increase the number of blood donors in this study. The participation in this study was voluntary, the participants did not receive any compensation and no stigmatizing questions were asked.

### Questionnaire development

For the development of the questionnaire, we conducted qualitative interviews with a total of 13 volunteers of which 5 were non-blood donors, 5 were blood donors and 3 were employees of the Bavarian Red Cross. Questions developed based on the information gathered in the qualitative interviews were then pre-tested with a subset of 22 volunteers and if necessary, adjustments of the questions were performed.

The survey consisted of questions on age, gender, current blood donor (options: yes/no), age at first blood donation and organ donor (options: yes/no). To be mentioned here is that participants were only classified as current blood donors, if they donated blood at least once within the last 18 months. In addition, we asked for pre-conditions (options: yes/no) and to rate the own health-related quality of life (HrQoL). The item used for measuring the HrQoL was based on the EQ-VAS, by using a scale of 0, worst imaginable HrQoL, to 100, best imaginable HrQoL [9]. Pre-conditions were defined as any existing cardiovascular, pulmonary, endocrinological, infectious or cancerous disease. Study participants were also asked, if they have blood donors within family and friends (options: none, family, friends, *family and friends*), their reasons for donating or not donating blood and if they received any education on blood donation in school, vocational training or university. Additionally, participants were asked to rate their knowledge on blood donation by using a scale of 0, not at all informed, to 100, fully informed. Further questions were on anxieties related to blood donation and if they think that blood donations are associated with any physical benefits or disadvantages (options: none, benefits, disadvantage). Finally, we asked on weekly exercise behavior of participants with the possibility to choose no exercise, at least 2x for 30 min per week, at least 3x for 30 min per week or at least 5x for 30 min per week.

### Statistical analysis

Questionnaires which were finished by at least 85% and in which the question if the participant is current blood donor was answered, were included into the study. For description of characteristics of study participants, percentage of males, participants with pre-condition, and organ donors were calculated for the total population, blood donors and non-blood donors, respectively. In addition, means and corresponding standard deviations (SD) were calculated for age and HrQoL for all three populations, and mean age at first blood donation for current blood donors.

To identify variables characterizing blood donors and non-blood donors, we applied univariate analysis. Associations between blood donation status and age, HrQoL or knowledge on blood donation were analyzed using logistic regression model, respectively. If being blood donor was associated with the following variables, gender, pre-conditions, being an organ donor, exercise behavior, blood donors within family / friends, anxiety related to blood donation, knowledge on physical benefits / disadvantages from blood donation, and receiving education on blood donation was analyzed using Pearson’s chi-squared or Fishers’ exact test, respectively. For analyses using Pearson’s chi-squared or Fishers’ exact test, subgroup(s) of the variables were compared to one reference subgroup, which was the female population in terms of gender and the populations which have chosen the answer option no / none for the other mentioned variables. For each variable odds ratio (OR) and corresponding 95% confidence intervals (95% CI) for being blood donor were calculated and a p-value of 0.05 was considered statistically significant. Statistical analysis was performed using JASP software package [10].

## Results

### Characteristics of study participants

A total of 682 participants with a mean age of 33.4 and SD of 12.0 years were included into the study (Table 1). Of these, 186 participants were blood donors (mean age 31.8 SD 10.8 years), and 496 were non-blood donors (mean age 33.9 SD 12.4 years). Percentage of male participants was 28.3%, 33.9% and 26.2% for total population, blood donors and non-blood donors, respectively. Number of organ donors was higher in blood donors (71.5%) compared to non-blood donors (52.4%), whereas the number of participants with pre-conditions was higher in non-blood donors (27.8% vs. 14.0%). Mean HrQoL was 80.1 (SD 16.1) in blood donors, 72.9 (SD 19.9) in non-blood donors and 74.8 (SD 19.2) in total population. The mean age of current blood donors at first blood donation was 21.8 (SD 6.1) years.

### Sociodemographic factors and knowledge influencing blood donors

Full analysis of variables characterizing blood donors and non-blood donors is shown in Table 2. The analysis indicated that increasing age was associated with a significant lower probability of being blood donor (OR: 0.99 [95% CI: 0.97; 1.00] *p*=0.043), whereas increasing knowledge on blood donation (OR: 1.04 [95% CI: 1.03; 1.05] *p*≤0.001) and increasing HrQoL (OR: 1.02 [95% CI: 1.01; 1.03] *p*≤0.001) were significantly associated with a higher likelihood of being blood donor, respectively.

Organ donors (OR: 2.78 [95% CI: 1.58; 3.28] *p*≤0.001) and study participants who know that blood donation is associated with physical benefits (OR: 3.07 [95% CI: 2.12; 4.45] *p*≤0.001) indicated significantly higher numbers of blood donors, respectively. In addition, blood donors within family (OR: 2.82 [95% CI: 1.32; 6.03] *p*=0.006) or friends (OR: 2.44 [95% CI: 1.24; 4.76] *p*=0.008) was also significantly associated with higher proportion of blood donors. Nevertheless, strongest factor for the probability of being blood donor was *blood donors within family and friends* (OR: 5.05 [95% CI: 2.63; 9.70] *p*≤0.001). In contrast, participants with pre-conditions were less frequent blood donors compared to participants without pre-conditions (OR: 0.42 [95% CI: 0.27; 0.67] *p*≤0.001). In particular, participants with anxiety related to blood donation were significantly less likely to be blood donors compared to participants without anxiety (OR: 0.11 [95%: 0.05; 0.21] *p*≤0.001) (Table 2).

Gender, education on blood donation in school, at vocational training or university as well as the number of exercises per week indicated no significant impact on the blood donation status (Table 2), respectively. But to be mentioned here is that male participants were non-significantly more likely to be blood donors (OR: 1.43 [95% CI: 1.00; 2.06]) with a close to significance *p*-value of 0.051.

### Personal reasons for blood donation

**Figure 1:**
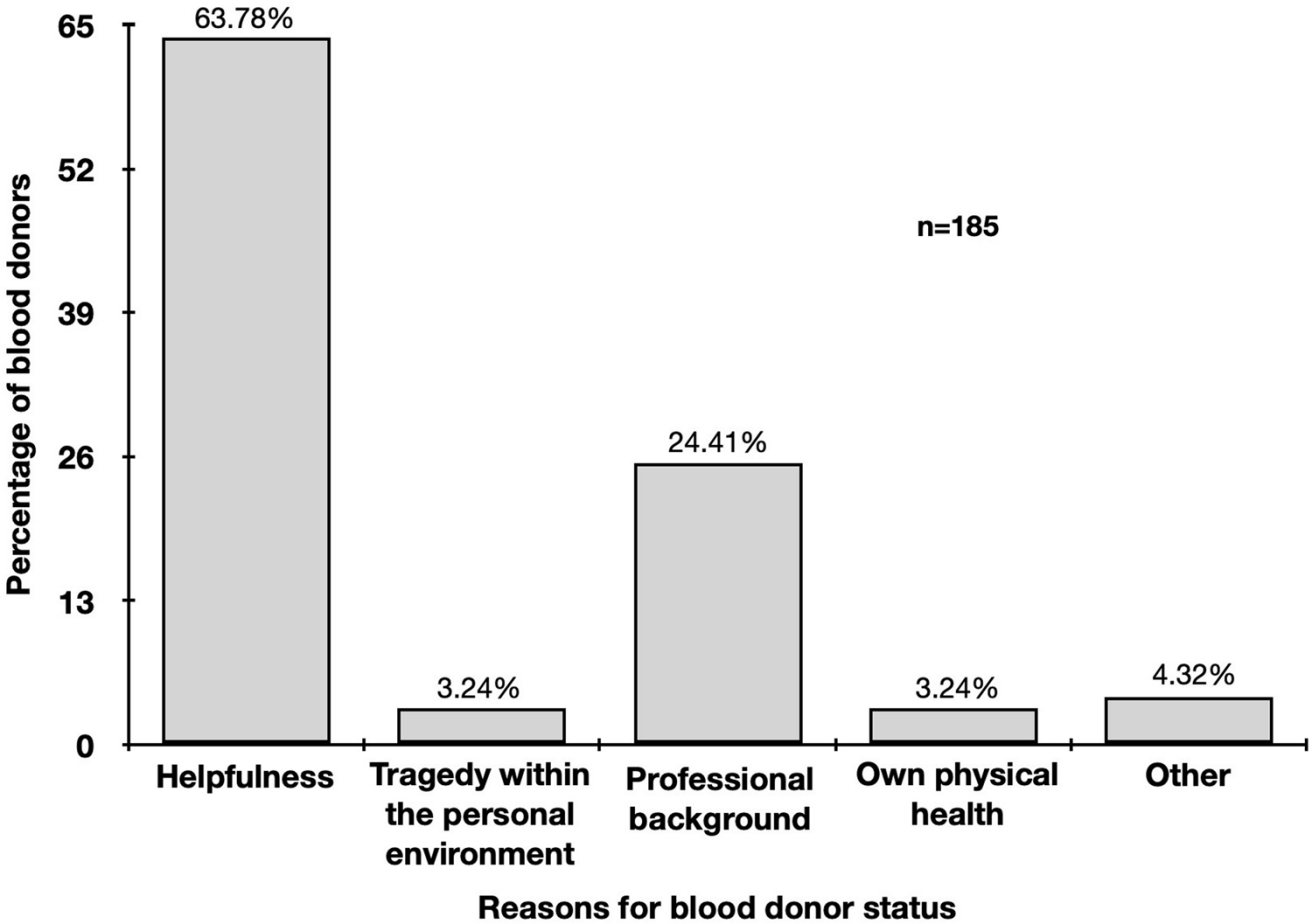
Reasons for blood donors to donate blood

**Figure 2:**
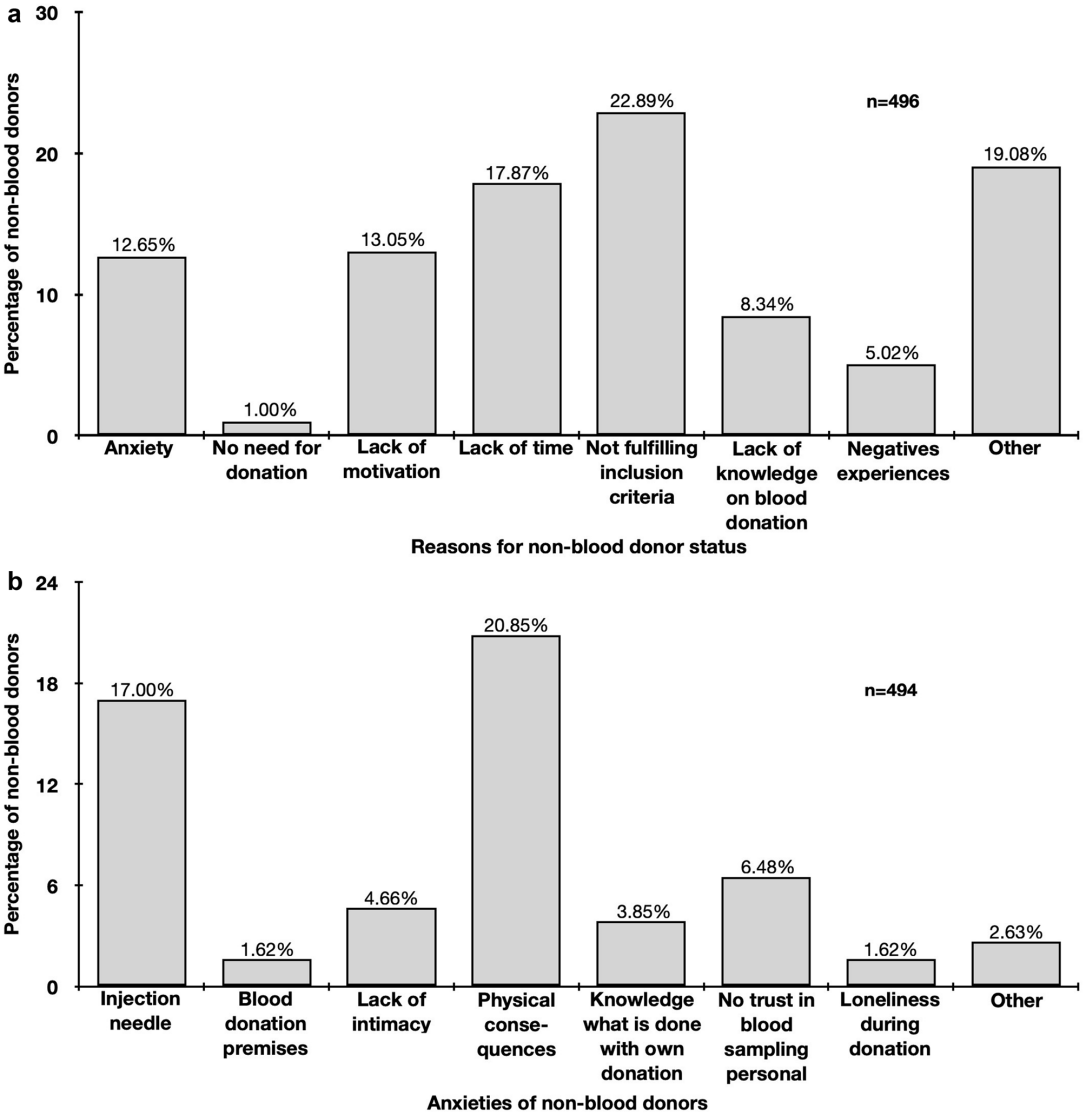
Reasons for non-blood donors for not donating blood (**a)** and anxieties of non-blood donors related to blood donation (**b)**. Multiple responses were allowed

Blood donors mentioned helpfulness (63.78%), professional background (24.41%), own physical health (3.24%), and tragedy within the personal environment (3.24%) as most frequent reasons to donate blood (Fig. 1). In contrast, not fulfilling the inclusion criteria (22.89%), other (19.08%), lack of time (17.87%), lack of motivation (13.05%), and anxiety (12.65%) were main causes to not donate blood (Fig. 2a). Most frequently mentioned reasons among *other* were pregnancy or breastfeeding (women only: 15.92%) and own health (both gender: 40.00%). Fear of physical consequences after blood donation (20.85%), injection needle (17.00%) and no trust in blood sampling personal (6.48%) were most frequently stated anxieties of non-blood donors (Fig. 2b).

**Figure 3:**
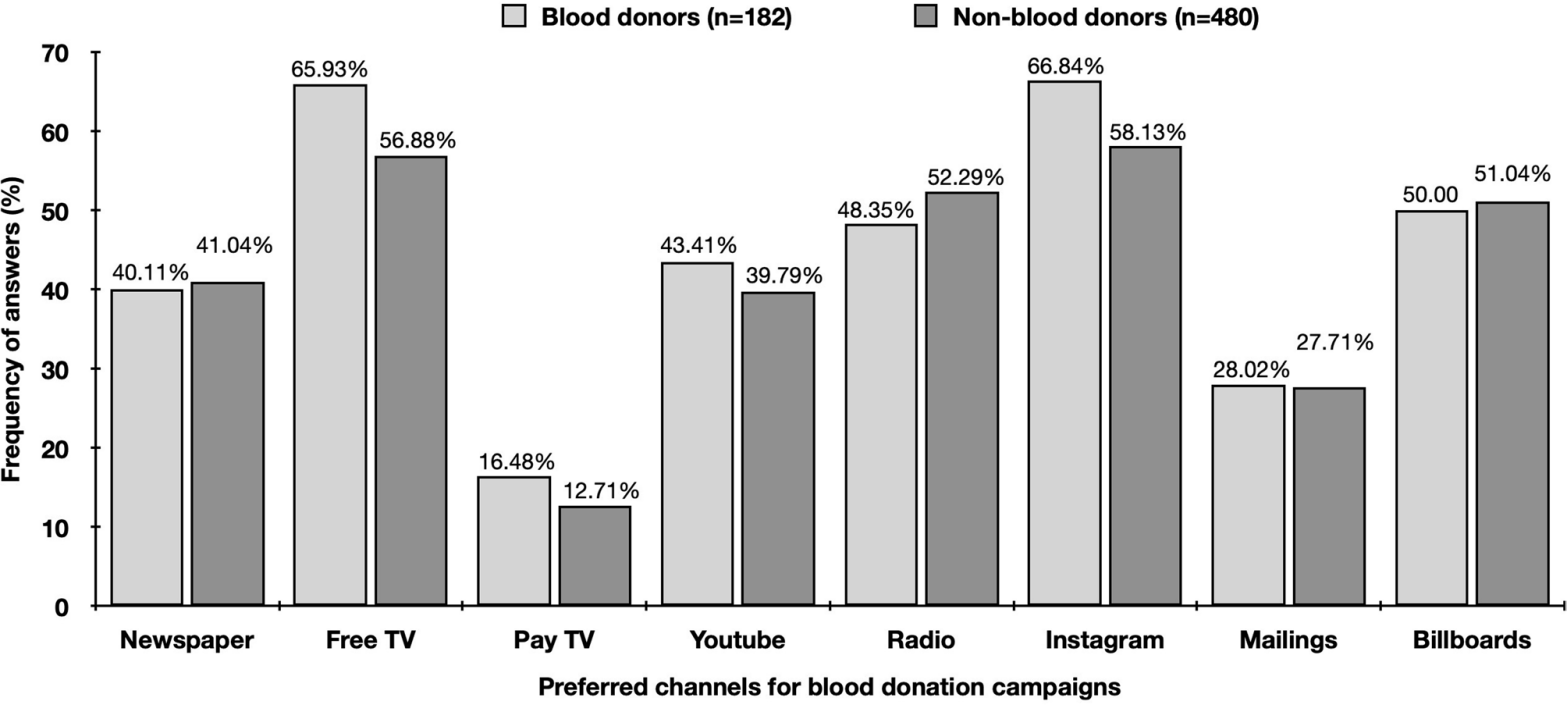
Preferred channels to promote blood donation campaigns. Multiple responses were allowed

Preferred channels which should be used for blood donation campaigns were comparable between blood donors and non-blood donors (Fig. 3). Based on the answers, Instagram (blood donors: 66.84% / non-blood donors: 58.13%), free TV (blood donors: 65.93% / non-blood donors: 56.88%), billboards (blood donors: 50.00% / non-blood donors: 51.04%) and radio (blood donors: 48.35% / non-blood donors: 52.29%) should be predominantly used to promote blood donation.

## Discussion

The results of this study indicate factors influencing the blood donation status in Germany as well as anxieties and reasons related to be (non-)blood donor. Strongest variable negatively influencing blood donation status was anxiety, whereas blood donors within family and friends was strongest variable to positively influence blood donation status.

In contrast to previous studies showing that women are more likely to be blood donors [6, 7], males were non-significantly more likely to be blood donors in the present study. The discrepancy compared to other studies might be related to the mean age of 31.8 years of the participants in this study. This mean age coincides with the mean age of women giving birth in Germany (31.5 years) according to the data of the Federal Statistical Office [11]. Additionally, up to 16% of female non-blood donors stated that pregnancy and breastfeeding is preventing them from donation. The present study also shows that the own health status is a strong determinant for blood donation status. Study participants with pre-conditions and lower HrQoL were less likely to be blood donors (Table 2). Similar findings were reported by Suen et al. (2020) and regarding satisfaction with the own health also by Studte et al. (2018) [6, 7].

Important and in line with previous data is that organ donors are more likely to be blood donors [6]. A study by Ferguson et al. (2012) identified that in contrast to non-blood donors, blood donors are more often altruistically motivated and satisfied by helping others (warm glow giving) [12]. The results of the present study reveal that more than 60% of blood donors stated helpfulness as reason for being blood donor. Therefore, it is assumable that organ donors are driven by similar reasons and it is not surprising that the number of blood donors is higher among organ donors compared to non-organ donors. Nevertheless, focusing on organ donors for the recruitment of new blood donors could be a strategy to increase the number of blood donations. This could be realized by joint organ and blood donor campaigns.

Further potential possibilities to recruit new blood donors are within the personal environment of current donors, since having blood donors within family and friends was strongly associated with being blood donor. Study participants with blood donors within family, friends or both family and friends were 2.8, 2.4 and 5.1 times more likely to be blood donors, respectively. Future blood donation campaigns should focus more on the personal environment of blood donors and might even engage current blood donors into campaigns by providing them tailored information material for their relatives and / or friends. In addition, and due to the fact that blood donors had an average age of 21.8 years at first blood donation, social media channels such as Instagram could be more intensively used for the promotion of blood donation campaigns. Especially, since 58.13% of non-blood donors mentioned Instagram and 39.79% Youtube as preferred channel for the promotion of blood donation.

Based on our data, education on blood donation is highly important. Unsurprisingly, participants with less knowledge on blood donation were more often non-blood donors. In contrast, participants knowing that blood donation is associated with health benefits were more likely to be blood donors. Of course, one has to keep in mind that blood donors have more knowledge due to their experiences with blood donation. Nevertheless, these findings on the influence of knowledge on blood donation status are supported by the study of Suen et al. (2020) from Hong Kong with a total of 542 participants [6]. Education is important to reduce anxieties, to educate that blood donation is not linked to tremendous physical consequences and to gain more trust in blood donation services. To be mentioned here is, that data from Italy indicates that blood donors have a lower risk for hospitalization, infectious disease or diseases related to the endocrine and blood system [13], respectively. A study from Finland showed that blood donors reveal an 88% lower risk of myocardial infarction compared to non-blood donors [14]. Examples for known negative physical effects related to blood donation are syncope, local bleeding, feeling tired, vertigo / dizziness or a diminished physical capacity [15, 16]. Nevertheless, positive physical effects of blood donation could be stronger integrated into campaigns in Germany. A potential focus of campaigns might be set on the potentially positive effects of blood donation on cardiovascular (CV) diseases, since CV diseases were the main cause of death in Germany in 2018 [17].

Interestingly, anxiety was strongly associated with being non-blood donor (Table 2), but anxiety was not the most frequently mentioned reason to not donate blood (Fig. 2a). We assume that this is due to the high number of study participants stating not fulfilling the criteria, having pre-conditions or a lack of time as reason for not donating blood. These participants presumably never thought about anxieties related to blood donation, because these other reasons already prevent them from donating. A study from Pakistan revealed that people with adequate knowledge were not donating blood if there is a lack of facilities and approaches [18]. This also indicates that besides knowledge, the availability of blood donation services is important and almost 18% of non-blood donors mentioned they are not having enough time to donate blood.

It is known that several regions reported a decrease in blood donations during SARS-CoV-2 pandemic [19, 20]. Since the study period was during the pandemic, we cannot exclude that the pandemic had an influence on our study sample. Due to the fact that older people and people with comorbidities are more vulnerable to severe COVID-19 cases [21], it is likely that more young and healthier people were current blood donors and participated in our study. In addition, the cancellation of medical procedures or less injuries due to the lockdown reduced the demand on blood products [19, 20]. This in turn might have influenced the blood donation behavior of potential study participants. Therefore, the previously mentioned aspects could have led to a recruitment bias and an influence on our analysis.

Despite the limitation related to the SARS-CoV-2 pandemic this study has further limitations, which will be discussed below.

First, the study was performed using an online survey and in terms of truthfulness of the answers we had to rely on the participants.

Second, the snowball system might have biased the recruitment of study participants. People in social environments may indicate similar characteristics, which in turn could have had an impact on the univariate analyses. Nevertheless, to recruit an adequate number of blood donors the access to the survey was also distributed using the Bavarian Red Cross.

Third, the mean age of the participants was relatively low with 33.4 years and therefore, the results might not represent the whole age range of people allowed to donate blood.

Fourth, our study only included a limited number of potential factors influencing blood donation status. More and broader studies are needed to obtain a complete overview of factors impacting the blood donation status.

Fifth, to assess HrQoL and knowledge of study participants on blood donation numerical scales were used, which provide less information compared to other instruments. Nevertheless, rating scales can be answered within a minimum of time and they are frequently used to assess HrQoL or severity of pain [9, 22].

## Conclusions

This study reveals that family and friends and being an organ donor are strong drivers to become blood donor. In addition, blood donors indicated more knowledge on blood donation and in particular on the physical benefits of donating blood. In contrast, anxiety (e. g. on physical consequences) and pre-conditions are strong drivers to be a non-blood donor. Based on our data, future blood donation campaigns could strongly consider and implement the following aspects: (1) blood donors should be involved into campaigns, e. g. by providing them special information materials to recruit their relatives and friends, (2) materials should explain potential effects of blood donations on physical health, but in particular provide more knowledge on potential physical benefits, (3) campaigns need to have a stronger focus on young adults below 25 years and (4) on social media channels, respectively.

**Table 1:** Characteristics of study participants

Characteristics	Blood Donors		Total (n=682)
	Yes (n=682)	No (n=496)	
Gender male - n (%)	63 (33.9%)	130 (26.2%)	193 (28.3%)
Age - mean (SD)	31.8 (10.8)	33.9 (12.4)	33.4 (12.0)
Organ donors - n (%)	133 (71.5%)	260 (52.4%)	393 (57.6%)
Age at first blood donation - mean (SD)	21.8 (6.1)	-	-
With precondition - n (%)	26 (14.0%)	138 (27.8%)	164 (24.1%)
HrQoL - mean (SD)	80.1 (16.1)	72.9 (19.9)	74.8 (19.2)

**Table 2:** Factors influencing blood donation status

Variable	Subgroups	OR (95% CI)	p-Value
Age	By one year age increase	0.99 (0.97; 1.00)	*p*=0.043
HrQoL	By one unit HrQoL increase	1.02 (1.01; 1.03)	*p*≤0.001
Knowledge in blood donation	By one unit knowledge increase	1.04 (1.03; 1.05)	*p*≤0.001
Gender	(1) Female, (2) Male	(1) Reference, (2) 1.43 (1.00; 2.06)	*p*=0.051
Pre-conditions	(1) No, (2) Yes	(1) Reference. (2) 0.42 (0.27; 0.67)	*p*≤0.001
Organ donor	(1) No, (2)Yes	(1) Reference, (2) 2,78 (1.58; 3.28)	*p*≤0.001
Exercises / week	(1) None, (2) Min. 2x 30 min / week, (3) Min. 3x 30 min / week, (4) Min. 5x 30 min / week	(1) Reference (2) 1.25 (0.83; 1.89), (3) 1.25 (0.79; 1.99), (4) 0.99 (0.55; 1.80)	*(2) p*=0.292, (3) *p*=0.347, (4) *p*=0.984
Blood donors within family / friends	(1) None, (2) Family, (3) Friends, (4) Family and friends	(1) Reference, (2) 2.82 (1.32; 6.03), (3) 2.44 (1.24; 4.76), (4) 5.05 (2.63; 9.70)	*(2) p*=0.006, (3) *p*=0.008, (4) *p*≤0.001
Anxiety	(1) No, (2) Yes	(1) Reference, (2) 0.11 (0.05; 0.21)	*p*≤0.001
Physical benefits /disadvantages from blood donation	(1) None, (2) Yes, benefits, (3) Yes, disadvantages	(1) Reference, (2) 3.07 (2,12; 4,45), (3) 0.63 (0.30; 1,33)	*(2) p*≤0.001, (3) *p*=0.224
Education on blood donation	(1) None, (2) In school, (3) During vocational training, (4) At university	(1) Reference, (2) 1.63 (0.83; 3.22), (3)1.16 (0.55; 2.44), (4) 1.49 (0.24; 6.77)	*(2) p*=0.155, (3) *p*=0.693, (4) *p*=0.699
